# Functional Analysis of the Superfamily 1 DNA Helicases Encoded by *Mycoplasma pneumoniae* and *Mycoplasma genitalium*

**DOI:** 10.1371/journal.pone.0070870

**Published:** 2013-07-23

**Authors:** Silvia Estevão, Helga U. van der Heul, Marcel Sluijter, Theo Hoogenboezem, Nico G. Hartwig, Annemarie M. C. van Rossum, Cornelis Vink

**Affiliations:** Laboratory of Pediatrics, Pediatric Infectious Diseases and Immunity, Erasmus MC-Sophia Children’s Hospital, Rotterdam, The Netherlands; Saint Louis University, United States of America

## Abstract

The DNA recombination and repair machinery of *Mycoplasma pneumoniae* is composed of a limited set of approximately 11 proteins. Two of these proteins were predicted to be encoded by neighboring open reading frames (ORFs) MPN340 and MPN341. Both ORFs were found to have sequence similarity with genes that encode proteins belonging to the DNA helicase superfamily 1 (SF1). Interestingly, while a homolog of the MPN341 ORF is present in the genome of *Mycoplasma genitalium* (ORF MG244), MPN340 is an *M. pneumoniae*-specific ORF that is not found in other mycoplasmas. Moreover, the length of MPN340 (1590 base pairs [bp]) is considerably shorter than that of MPN341 (2148 bp). Examination of the MPN340-encoded amino acid sequence indicated that it may lack a so-called 2B subdomain, which is found in most SF1 DNA helicases. Also, the MPN340-encoded amino acid sequence was found to differ between subtype 1 strain M129 and subtype 2 strain FH at three amino acid positions. Both protein variants, which were termed PcrA^s^_M129_ and PcrA^s^_FH_, respectively, as well as the MPN341- and MG244-encoded proteins (PcrA*_Mpn_* and PcrA*_Mge_*, respectively), were purified, and tested for their ability to interact with DNA. While PcrA*_Mpn_* and PcrA*_Mge_* were found to bind preferentially to single-stranded DNA, both PcrA^s^_M129_ and PcrA^s^_FH_ did not demonstrate significant DNA binding. However, all four proteins were found to have divalent cation- and ATP-dependent DNA helicase activity. The proteins displayed highest activity on partially double-stranded DNA substrates carrying 3′ single-stranded extensions.

## Introduction

*Mycoplasma pneumoniae* and *Mycoplasma genitalium* are genetically closely related human pathogens that are classified within the bacterial class of *Mollicutes*. These bacteria represent the smallest known self-replicating organisms. It is generally accepted that the *Mollicutes* have evolved from a Gram-positive ancestor by a gradual, but significant, reduction in genome size and gene content. Consequently, the genomes of *M. pneumoniae* (strain M129) and *M. genitalium* (strain G37) are small in comparison to those of bacteria from other classes (816 kb and 580 kb, respectively) [1]–[3]. Also, various biochemical pathways in these bacteria are either lacking or orchestrated by a limited set of enzymes. Among the mycoplasmal pathways that appear to be significantly less convoluted than that of model bacterium *Escherichia coli* is the DNA recombination and repair (DRR) system. In spite of the important functions that this system may have in both replication and (antigenic) variation of pathogenic mycoplasmas (see Vink et al. for a recent review [4]), it is yet unknown how DRR is actually achieved by these ‘minimal’ bacteria. From *in silico* analyses of mycoplasma genomes it was predicted that they possess the most compact set of recombination-associated genes of all known bacteria, consisting of approximately 11 genes [5], [6]. These genes have the capacity to code for proteins putatively involved in homologous DNA strand transfer (RecA and SSB), Holliday junction (HJ) branch migration and resolution (RuvA, RuvB and RecU), nucleotide excision repair (UvrA, UvrB, UvrC, and PcrA) and base excision repair (Nfo endonuclease IV). In comparison to other bacteria, however, *M. pneumoniae* and *M. genitalium* appear to lack homologs of several other enzymes and/or enzymatic pathways involved in DRR. Specifically, enzymes such as LexA and others related to the SOS response are absent. In addition, both mycoplasmas lack RecBCD, AddAB, RecQ, RecJ and RecFOR [5], [6]. Nonetheless, despite the apparent limitations of their DRR machineries, homologous DNA recombination events were found to occur in both *M. pneumoniae* and *M. genitalium* [7]–[11].

To understand how DRR is executed and regulated in *M. pneumoniae* and *M. genitalium*, we have previously initiated the characterization of the putative recombination proteins from these species. As of yet, the in vitro activities have been determined of the SSB protein from *M. pneumoniae* [12] and the RecA [13], RecU [14], [15], RuvB [16] and RuvA proteins [17], [18]) from both *M. pneumoniae* and *M. genitalium*. Surprisingly, in spite of the high level of sequence conservation between these bacteria, significant differences were found in the activities of some of their orthologous recombination proteins. Most notably, *M. pneumoniae* was found unable to express a functional RecU protein, whereas *M. genitalium* RecU is a very potent Holliday junction resolvase [14]. In addition, functional differences were noted between the RuvA orthologs and RuvB orthologs from *M. pneumoniae* and *M. genitalium* [16], [17].

To increase the understanding of the functionality of the ‘minimal’ DRR machinery of the pathogenic mycoplasmas, we have focused our attention on the *M. pneumoniae* and *M. genitalium* ORFs that share sequences with genes encoding PcrA/Rep/UvrD-like DNA helicases. These enzymes belong to the superfamily 1 (SF1) of DNA helicases, and function as helicases or nucleic acid translocases in almost every aspect of the nucleic acid metabolism, such as DNA repair and the replication of specific plasmids [19]–[24]. While Rep and UvrD are found in Gram-negative bacteria, PcrA is the SF1 DNA helicase that is encoded by bacteria belonging to the *Firmicutes* and *Mollicutes* classes. The biological relevance of the PcrA helicases has previously been demonstrated for two Gram-positive species, i.e. *Bacillus subtilis* and *Staphylococcus aureus.* The PcrA orthologs from these species (PcrA*_Bsu_* and PcrA*_Sau_*, respectively) were both shown to be essential for cell growth [25]–[27].

Although most *Firmicutes* and *Mollicutes* species (including *M. genitalium*) possess a single *pcrA* gene, *M. pneumoniae* harbors two neighboring ORFs encoding PcrA homologs, i.e. MPN340 and MPN341. As only the latter ORF is conserved between *M. pneumoniae* and *M. genitalium*, MPN340 represents an *M. pneumoniae*-specific ORF. In this study, we have characterized and compared the in vitro activities of all PcrA(-like) proteins encoded by both *M. pneumoniae* and *M. genitalium*.

## Materials and Methods

### Cloning of the MPN340, MPNE_0394, MPN341 and MG244 ORFs

Bacterial genomic DNA was purified from cultures of *M. genitalium* strain G37 (ATCC® no. 33530™) and *M. pneumoniae* strains M129 (ATCC® no. 29342™) and FH (ATCC® no. 15531™), using previously described procedures [12]. Before cloning of ORFs MPN340 and MPNE_0394 from *M. pneumoniae* strains M129 and FH, respectively, a TGA codon within these ORFs was changed into a TGG codon using a PCR-based mutagenesis method [14]. In this procedure, the products from two separate PCRs (one with primers pET-Fw and Mutation-Rv, and another with primers Mutation-Fw and pET-Rv; supporting Table S1) were mixed, and subjected to a PCR with primers pET-Fw and pET-Rv. The resulting PCR product was digested with *Nde*I and *Bam*HI (for which cleavage sites are present within the sequences of pET-Fw and pET-Rv, respectively), and ligated into *Nde*I- and *Bam*HI-digested vectors pET-11c and pET-16b (Novagen). The resulting plasmids were used as templates in PCRs with primers pMAL-c_Fw and pMAL-c_Rv. The amplified fragments were then digested with *Eco*RI and *Pst*I and cloned into *Eco*RI-and *Pst*I-digested vector pMAL-c (New England Biolabs).

Both MPN341 and MG244 were found to contain five TGA codons. These codons were changed into TGG codons in a similar fashion as described above, using a set of overlapping PCR products. These products were generated using the primers listed in supporting Table S1. For MPN341, overlapping PCR fragments were generated with the following primer pairs: (i) 341pETfw and 341Mut1rv, (ii) 341Mut1 and 341Mut2rv, (iii) 341Mut2 and 341Mut3rv, (iv) 341Mut3 and 341Mut4rv, (v) 341Mut4 and 341Mut5rv, and (vi) 341Mut5fw and 341pETrv. The outer primer pair 341pETfw and 341pETrv was employed to amplify the complete, modified MPN341 ORF. The resulting PCR product was digested with *Nde*I and *Bam*HI and ligated into *Nde*I- and *Bam*HI-digested vectors pET-11c and pET-16b. The resulting plasmids were used as templates in PCRs with primers 341pMALcfw and 341pMALcrv. The amplified fragments were then digested with *Xba*I and *Pst*I and cloned into *Xba*I-and *Pst*I-digested vector pMAL-c.

ORF MG244 was modified and cloned in a similar fashion as described above for MPN341, using the MG244-specific primers listed in Table S1. Like the other three ORFs, the modified MG244 ORF was cloned in vectors pET-11c, pET-16B and pMAL-c. The pET-11c- and pET-16B-derived plasmids were employed for expression of native and poly-histidine (H_10_)-tagged proteins, respectively, in *Escherichia coli* strain BL21(DE3)pLysS. The pMAL-c-derived plasmids were used for the expression of maltose-binding protein (MBP)-fused proteins in *E. coli* strain XL1-Blue. The integrity of all DNA constructs used in this study was checked by dideoxy sequencing, as described before [13].

### Generation of Plasmids Encoding Point Mutants of PcrAsM129

Expression constructs encoding point mutants of PcrA^s^_M129_ (K29R and K29A) were constructed using a mutagenesis procedure similar to that described above for modification of the TGA codons. The primers used for generation of the construct encoding mutant K29R were 340FW_K>R and 340RV_K>R (Table S1). The construct encoding K29A was generated using primers 340FW_K>A and 340RV_K>A. The final PCR products were cloned into vector pMAL-c.

### Expression and Purification of the PcrA Proteins

The proteins encoded by MPN340, MPNE_0394, MPN341 and MG244 were expressed in *E. coli* as native proteins, and as H_10_- and MBP-tagged proteins. The native and H_10_-tagged proteins were found to be expressed exclusively in an insoluble form (under various culturing conditions). However, the proteins could readily be expressed and purified as MBP-tagged proteins. These proteins, as well as negative control protein MBP-β-galactosidase-α (hereafter named MBP), were purified using a previously described procedure [28].

### SDS-Polyacrylamide Gel Electrophoresis (SDS-PAGE) and Mass Spectrometry

Proteins were analyzed by SDS-PAGE, as described by Laemmli [29]. The gels were stained with Coomassie brilliant blue (CBB), destained in 40% methanol/10% acetic acid, and recorded using a GelDoc XR system (Bio-Rad). Digital images were processed using Quantity One® 1-D Analysis Software (Bio-Rad). Mass spectrometry of proteins was performed by matrix-assisted laser desorption/ionisation-time of flight (MALDI-TOF) mass spectrometry, using an Ultraflex MALDI-TOF/TOF mass spectrometer (Bruker Daltonics), as described previously [30].

### DNA Substrates

**Figure 1:**
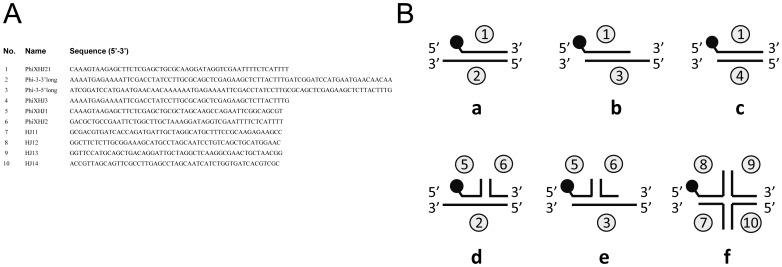
Sequences and structures of the DNA substrates used in this study. (A) Numbers, names and sequences of the oligonucleotides that were used to generate the DNA substrates shown in (B). (B) Schematic structures of the DNA substrates. Oligonucleotides are indicated as numbered lines. The numbers (in circles) correspond to the numbers of the oligonucleotides shown in (A). Substrates ‘a’ and ‘d’ contain a 3′ 24-nt ss extension, substrates ‘b’ and ‘e’ contain a 5′ 24-nt ss extension, and substrates ‘c’ and ‘f’ are blunt-ended. The black dots represent 6-FAM labels at the 5′ end of the oligonucleotides.

The sequences and structures of the oligonucleotide substrates that were used in the DNA binding and DNA helicase experiments are shown in Fig. 1. In each substrate, a single oligonucleotide strand was labeled at its 5′ terminus with a fluorescent (6-FAM) group.

### Electrophoretic Mobility Shift Assay (EMSA)

Binding of the PcrA proteins to various DNA substrates was carried out in 10-µl volumes and included 20 mM Tris-HCl pH 7.5, 1 mM DTT, 50 ng/µl BSA, 8 nM of substrate DNA and varying concentrations of protein. After incubation for 15 min at room temperature, 1 µl was added of a solution containing 40% glycerol and 0.25% bromophenol blue. Then, the reaction mixtures were electrophoresed through 6% polyacrylamide gels in 1×TBE buffer (90 mM Tris, 90 mM boric acid, 2 mM EDTA). Following electrophoresis, the polyacrylamide gels were analyzed by fluorometry, using a Typhoon Trio™ 9200 Variable Mode Imager (GE Healthcare) in combination with the Typhoon Scanner Control v4.0 software (Amersham Bioscience). Images were processed using Quantity One® 1-D Analysis Software [31].

### DNA Helicase Assays

DNA helicase assays were performed similarly as described before [16]. Standard reactions (10 µl) contained 20 mM Tris-HCl pH 7.5, 1 mM DTT, 50 ng/µl BSA, 2.5 mM MgCl_2_, 1 mM ATP, either 4 or 8 nM of substrate DNA (Fig. 1), and various concentrations of proteins. Reactions were carried out for 5 min at 37°C, after which the reactions were terminated by addition of 1 µl of Termination Mix (100 mM Tris-HCl pH 7.4, 5% SDS, 0.2 M EDTA) and 1 µl of Proteinase K (at 10 mg/ml). After deproteinization (15 min at 37°C), 1.5 µl of loading dye (40% glycerol, 0.25% bromophenol blue) was added to the samples, which were subsequently electrophoresed through native 12% polyacrylamide/1×TBE (90 mM Tris, 90 mM boric acid, 2 mM EDTA) mini-gels. Gels were analyzed by fluorometry, as described above.

### ATPase Assay

ATPase activity was determined in the presence or absence of φX174 virion DNA (at a final concentration of 1.5 nM) using a β-nicotinamide adenine dinucleotide reduced form (NADH)-coupled assay on a VersaMax Tunable Microplate Reader (Molecular Devices), as described before [13], [32]. The ATP turnover rates were calculated from the equation: ATPase rate (ATP×min^−1^) = -dOD_340_/dt (OD/min)×K_path_^−1^ (mol/OD)×mol^−1^ PcrA protein, where K_path_ is the molar absorption coefficient for NADH for a given optical pathlength [33]. The rates were corrected for background NADH decomposition of controls performed without protein.

## Results

### *M. pneumoniae* and *M. genitalium* Encode PcrA Homologs

The genome of *M. pneumoniae* strain M129 contains two neighboring ORFs, MPN340 and MPN341, which were both annotated as genes encoding UvrD-like helicases [1], [2]. However, these ORF differs significantly in size; while MPN340 has a length of 1,590 bp, MPN341 measures 2,148 bp. In contrast to *M. pneumoniae*, *M. genitalium* only contains a single gene putatively encoding a UvrD-like protein [3]. This gene, MG244, was suggested to represent the ortholog of *M. pneumoniae* MPN341. To investigate the relationship between these ORFs and similar sequences in the GenBank sequence database, their encoded amino acid sequences were subjected to protein BLAST analysis using the blastp algorithm (http://blast.ncbi.nlm.nih.gov/Blast.cgi?PAGE=Proteins). The MPN340-encoded amino acid sequence displayed the highest similarity with the sequence encoded by ORF MPNE_0394, which is the MPN340 counterpart of *M. pneumoniae* strain FH (99% identity; Table 1). These sequences were found to differ in only three amino acid residues. Lower similarities were found with the sequences encoded by MPN341 (47% identity) and MG244 (45% identity). Relatively high similarity scores were also found with PcrA sequences from Gram-positive bacteria, including *Lactobacillus salivarius* and *Staphylococcus aureus*. To address their relatively strong sequence similarity with PcrA(-like) proteins, the MPN340-encoded proteins from strains M129 and FH were named PcrA^s^_M129_ and PcrA^s^_FH_, respectively, in which the superscript ‘s’ (short) indicates the relatively short size of these proteins as opposed to the MPN341-encoded protein. The latter protein does not differ in sequence between strains M129 and FH and was termed PcrA*_Mpn_*. The *M. genitalium* ortholog of PcrA*_Mpn_* was designated PcrA*_Mge_*. The sequences of these proteins, as well as those from the PcrAs of *L. salivarius* and *S. aureus*, were included in a multiple sequence alignment (Fig. S1). The alignment demonstrated that the MPN340-, MPNE_0394-, MPN341- and MG244-encoded sequences each have features characteristic of proteins belonging to the SF1A group from the SF1 superfamily of DNA helicases [22]–[24], [34]. Most notably, these features include seven conserved protein motifs (motifs I, IA and II to VI) that may be involved either in the binding and hydrolysis of ATP or in the binding of DNA. Interestingly, in contrast to PcrA*_Mpn_* and PcrA*_Mge_*, PcrA^s^_M129_ and PcrA^s^_FH_ lack a counterpart of subdomain 2B, which is one of the four helicase subdomains that have previously been identified in the crystal structures of several PcrA/Rep/UvrD-like proteins, including PcrA from *Bacillus stearothermophilus* (PcrA*_Bst_*) and the Rep, UvrD and RecB proteins from *E. coli* [35]–[39]. As a consequence of the lack of a 2B subdomain, the theoretical molecular mass of PcrA^s^_M129_ and PcrA^s^_FH_ (60.5 kDa) is significantly lower than that of PcrA*_Mpn_* (83.5 kDa) and PcrA*_Mge_* (82.0 kDa).

**Table 1:** Amino acid sequence similarities (% identity) between the PcrA-like helicases from *M. pneumoniae* and *M. genitalium.*

ORF^a^	Predicted protein	PcrA^s^_M129_	PcrA^s^_FH_	PcrA*_Mpn_*	PcrA*_Mpn_*Δ2B^b^	PcrA*_Mge_*	PcrA*_Mge_*Δ2B^b^
**MPN340**	**PcrA^s^_M129_**	100%	99%	47%	44%	45%	43%
**MPNE_0394**	**PcrA^s^_FH_**	99%	100%	47%	44%	45%	43%
**MPN341**	**PcrA** ***_Mpn_***	47%	47%	100%	–	53%	–
**MPN341Δ2B**	**PcrA** ***_Mpn_*** **Δ2B**	44%	44%	–	100%	–	58%
**MG244**	**PcrA** ***_Mge_***	45%	45%	53%	–	100%	–
**MG244Δ2B**	**PcrA** ***_Mge_*** **Δ2B**	43%	43%	–	58%	–	100%

a The different ORFs are from *M. pneumoniae* strains M129 (MPN340 and MPN341) and FH (MPNE_0394), and from *M. genitalium* strain G37 (MG244).

b ‘PcrA*_Mpn_*Δ2B’ and ‘PcrA*_Mge_*Δ2B’ sequences represent PcrA*_Mpn_* and PcrA*_Mge_* sequences, respectively, from which the (predicted) subdomain 2B has been deleted.

**Figure 2:**
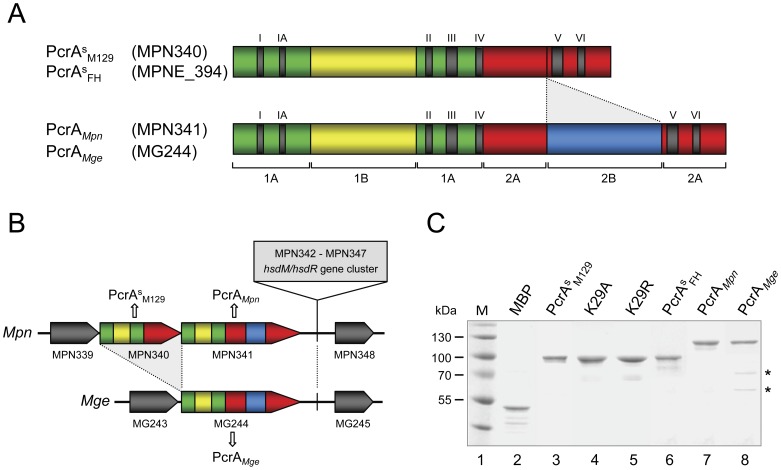
Predicted domain structure and purification of the PcrA-like proteins from *M. pneumoniae* and *M. genitalium*. (A) Schematic representation of the predicted (sub)domains (1A, 1B, 2A and 2B) and motifs (I, Ia and II to VI) of the PcrA-like proteins from *M. pneumoniae* and *M. genitalium*. The predictions are based on the multiple alignment shown in supporting Fig. S1, and on the crystal structures of PcrA/UvrD/Rep homologs [35], [60]. Each subdomain is indicated by a separate color. Counterparts of the predicted 2B domains from PcrA*_Mpn_* and PcrA*_Mge_* (in blue) are absent from the PcrA^s^_M129_ and PcrA^s^_FH_ proteins. (B) ORF structure of the genomes of *M. pneumoniae* (*Mpn*, at the top) and *M. genitalium* (*Mge*, at the bottom) in the region surrounding the ORFs encoding the PcrA-like proteins. All ORFs in these regions have the same orientation, i.e. 5′→3′ from left to right. The ORFs encoding the PcrA-like proteins are presented with the same color scheme as that of their encoded proteins in (A). Neighboring ORFs that are conserved between *M. pneumoniae* and *M. genitalium* are indicated in dark grey. The cluster of genes that is unique to *M. pneumoniae* (consisting of ORFs MPN342 to MPN347) is indicated at the top. (C) Purification of MBP, PcrA^s^_M129_, K29A, K29R, PcrA^s^_FH_, PcrA*_Mpn_* and PcrA*_Mge_*. K29A and K29R are point mutants of protein PcrA^s^_M129._ All proteins were purified as fusions to MBP by using the same protocol. Some of the protein preparations, including those of MBP and PcrA*_Mge_*, contained minor protein species that are smaller than the full-length proteins. These species are not contaminants, but breakdown products of the full-length proteins; this was demonstrated by MALDI-TOF analysis of the two protein species that are indicated with asterisks (*). Samples of the purified proteins (as indicated above the lanes) were analyzed by SDS-PAGE (10%) and Coomassie brilliant blue (CBB)-staining. The sizes of protein markers (lane 1; PageRulerTM Prestained Protein Ladder [Fermentas]) are shown on the left-hand side of the figure in kDa.

The structural differences between the PcrA^s^ proteins and the PcrA proteins are illustrated schematically in Fig. 2A. Clearly, the absence of the 2B subdomain from the PcrA^s^ proteins is the most significant difference between these proteins and other PcrA(-like) proteins. Although MPN340 and MPN341 are adjacent ORFs in the *M. pneumoniae* M129 genome (Fig. 2B), two major observations support the notion that the latter ORF is the ortholog of *M. genitalium* MG244. First, the 2B subdomain is conserved between MPN341 and MG244. Second, the amino acid sequence similarity between PcrA*_Mpn_* and PcrA*_Mge_* (53% identity; Table 1) is higher than the similarity between the PcrA^s^ proteins and PcrA*_Mge_* (45% identity). If the 2B domains are not considered in these sequence comparisons, the sequence similarity between PcrA*_Mpn_* and PcrA*_Mge_* is even higher (58% identity; Table 1), whereas the similarity between the PcrA^s^ proteins and PcrA*_Mge_* is somewhat lower (43% identity).

### Purification of the PcrA-like Proteins from *M. pneumoniae* and *M. genitalium*

To determine the characteristics of the PcrA-like proteins from both *M. pneumoniae* and *M. genitalium*, the proteins were expressed in *E. coli*, either in their native forms or fused to maltose-binding protein (MBP) or a poly-histidine (H_10_) tag. The native and H_10_-tagged versions of the proteins were either expressed at very low levels or in a solubility state that precluded their purification. In contrast, the MBP-tagged versions of the four proteins were expressed in a soluble form and could readily be purified using the same protocol for each protein [40]. Although these proteins carry an N-terminal tag, the use of this tag has several advantages. First, the activity of the proteins can be compared to that of a negative control protein (MBP-β-galactosidase-α [MBP]), which has been purified using the same method. Second, the amylose affinity-based purification protocol that is employed for the MBP-fused proteins is both efficient and rapid, which is beneficial to the proteins’ activity and stability [14]–[16], [40]–[42]. Third, the activities of the purified proteins (and mutants thereof; see below) can be compared directly, and are not influenced by differences in purification procedures. Moreover, other SF1A family members, such as the PcrA proteins from *Bacillus anthracis* (PcrA*_Ban_*) [43], [44], *Bacillus cereus* (PcrA*_Bce_*) [44], *Staphylococcus aureus* (PcrA*_Sau_*) [20], [45] and *Streptococcus pneumoniae* (PcrA*_Spn_*) [46], as well as the Rep protein from *E. coli* (Rep*_Eco_*) [47], were previously reported to be active as N-terminally tagged fusion proteins in vitro. We therefore anticipated that an N-terminal tag would not interfere in the analysis and comparison of the in vitro activities of the PcrA-like proteins from *M. pneumonia* and *M. genitalium*. The purified proteins, which will be referred to without the prefix ‘MBP’, were 90–95% pure (Fig. 2C). Some of the protein preparations, including those of MBP and PcrA*_Mge_* (Fig. 2C, lanes 2 and 8), contained minor products having a lower molecular mass than the full-length proteins. Such products are regularly observed for MBP fusions, particularly when these proteins are relatively large [14], [40], [42]. To investigate the nature of these products, the two minor species from the PcrA*_Mge_* preparation (indicated by the asterisks in Fig. 2C, lane 8), as well as the full-length protein, were excised from an SDS-polyacrylamide gel and subjected to MALDI-TOF mass spectrometry. In each of these protein species, amino acid sequences were identified that were contained within either MBP or PcrA*_Mge_* (data not shown) demonstrating that the minor, lower molecular mass species in the PcrA*_Mge_* preparation are the products of either premature translation termination or proteolytic breakdown of the MBP-fused PcrA*_Mge_*.

### DNA-binding Activity of the PcrA-like Proteins

**Figure 3:**
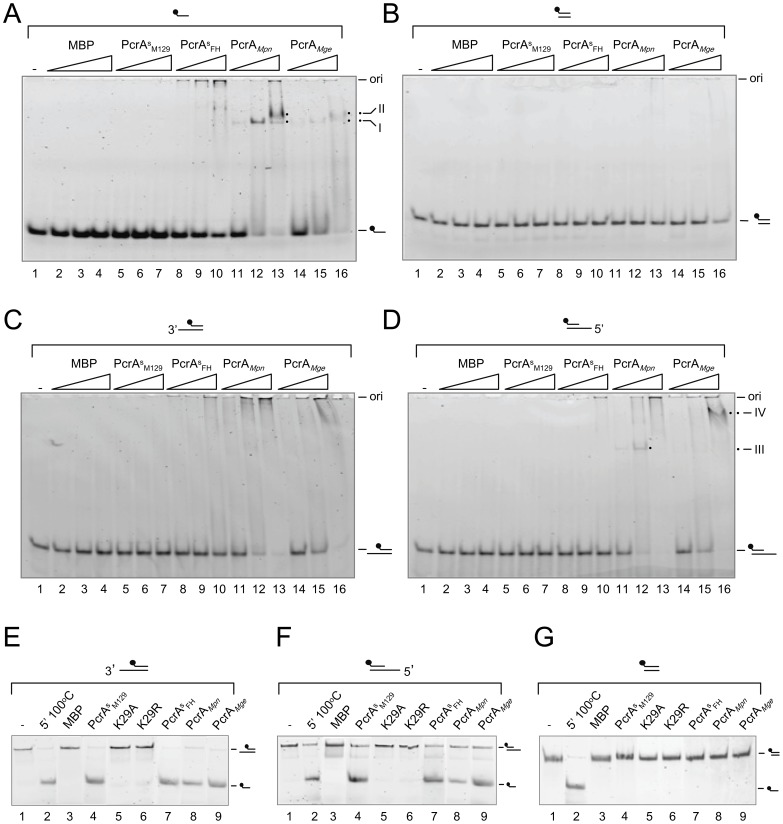
DNA-binding and DNA helicase activities of the PcrA proteins from *M. pneumoniae* and *M. genitalium*. (A) Binding of the PcrA proteins to 6-FAM-labeled oligonucleotide 1 was tested by EMSA. The PcrA proteins, as well as control protein MBP, were tested at 22 nM, 67 nM and 200 nM, respectively, as indicated from left to right by the triangles above the lanes. Protein was omitted from the reaction shown in lane 1 (‘−’). The positions of the free substrate and the position of protein-DNA complexes (I and II) are indicated at the right-hand side of the gel. Complex I and II are also pointed out by dots next to lanes 13 and 16. ‘ori’ indicates the origin of the gels. (B) Binding of the PcrA proteins and MBP to ds, blunt-ended substrate ‘c’. The experiment was performed similarly as described in (A). (C) Binding of the PcrA proteins and MBP to partially ds substrate ‘a’ (which contains a 3′ 24-nt ss extension). The experiment was executed similarly as described in (A). (D) Binding of the PcrA proteins and MBP to partially ds substrate ‘b’ (which contains a 5′ 24-nt ss extension). The experiment was done in a similar fashion as described in (A). (E) DNA helicase activity of the PcrA proteins on substrate ‘a’. The DNA helicase reactions were performed as described in Materials and Methods. Reactions were carried out in volumes of 10 µl and contained DNA substrate ‘a’ (at 4 nM) and either 0 nM (marked ‘−’, lane 1) or 50 nM of protein (as indicated above the lanes). After the reaction, the samples were deproteinized, electrophoresed through a native 12% polyacrylamide gel, and analyzed by fluorometry. The positions of the substrate and reaction products are indicated at the right-hand side of the gel. (F) DNA helicase activity of the PcrA proteins on substrate ‘b’. The experiment was done in a similar way as in (E). (G) DNA helicase activity of the PcrA proteins on substrate ‘c’. The experiment was performed as described in (E).

The DNA-binding properties of the PcrA-like proteins were investigated in an electrophoretic mobility shift assay (EMSA), using four different fluorescently labeled DNA substrates, i.e. a single-stranded (ss) oligonucleotide (oligonucleotide 1 from Fig. 1A), a double-stranded (ds), blunt-ended oligonucleotide (substrate ‘c’ from Fig. 1B), a ds oligonucleotide with a 3′ 24-nucleotide (nt) ss terminus (substrate ‘a’), and a ds oligonucleotide with a 5′ 24-nucleotide (nt) ss terminus (substrate ‘b’). As shown in Fig. 3A, both PcrA*_Mpn_* (lanes 11–13) and PcrA*_Mge_* (lanes 14–16) bound efficiently to the ss oligonucleotide in a protein concentration-dependent fashion. At PcrA*_Mpn_* concentrations of 22 nM (lane 11) and 67 nM (lane 12), a single protein-DNA complex (complex I) was observed. At 200 nM however, another, slower migrating complex (complex II) was seen, in addition to other complexes that were too large to enter the gel (lane 13). Although the PcrA*_Mge_* protein gave rise to similar protein-DNA complexes (lanes 14–16), these complexes were more diffuse than those formed with PcrA*_Mpn_*. Binding of PcrA*_Mpn_* and PcrA*_Mge_* was also observed to substrates carrying ss extensions (Fig. 3C and 3D). However, while PcrA*_Mpn_* and PcrA*_Mge_* formed discrete complexes with substrate ‘b’ (complex III and IV, respectively, in Fig. 3D), most of the complexes that were formed with substrate ‘a’ did not enter the gel (lanes 11–16). In contrast to the (partially) ss DNA substrates, the ds, blunt-ended substrate was bound very inefficiently by PcrA*_Mpn_* and PcrA*_Mge_* (Fig. 3B). Contrary to PcrA*_Mpn_* and PcrA*_Mge_*, PcrA^s^_M129_ did not demonstrate significant binding to any of the four DNA substrates. However, PcrA^s^_FH_ did show some complex formation with the ssDNA substrate (Fig. 3A, lanes 8–10), albeit that this activity was considerably lower than that observed for PcrA*_Mpn_* and PcrA*_Mge_*. The negative control protein, MBP, did not show binding to any of the DNA substrates (Fig. 3A–D, lanes 2–4).

### PcrA^s^_M129_, PcrA^s^_FH_, PcrA*_Mpn_* and PcrA*_Mge_* possess DNA Helicase Activity

To test the putative DNA helicase activities of the purified proteins, they were incubated with DNA substrates ‘a’, ‘b’ and ‘c’, in the presence of ATP and Mg^2+^ (Fig. 3E–G). Despite the inability of PcrA^s^_M129_ to bind to DNA in EMSA (as shown above), this protein was capable of unwinding both substrate ‘a’ (Fig. 3E, lane 4) and ‘b’ (Fig. 3F, lane 4). Similar activities were displayed by the other three PcrA-like proteins (Fig. 3E and 3F, lanes 7–9). However, these proteins did not display significant unwinding activity on blunt-ended substrate ‘c’ (Fig. 3G). As expected, negative control protein MBP did not show DNA unwinding activity on any of the three substrates (Fig. 3E–G, lane 3).

As additional (negative) control proteins, we purified two point mutants of PcrA^s^_M129_, which carry either a Lys to Arg mutation (in mutant K29R) or Lys to Ala mutation (in mutant K29A) at position 29 of the protein (Fig. 2C, lanes 4 and 5). Lys29 is an amino acid residue that is predicted to form an invariant and essential part of conserved motif I of PcrA^s^_M129_ (Fig. S1), and may be involved in nucleotide cofactor binding. As shown in Fig. 3E–G (lanes 5 and 6), both K29R and K29A did not display significant DNA unwinding activity on any of the DNA substrates used in this study. This result underlines the importance of amino acid residue Lys29 in the DNA unwinding activity of PcrA^s^_M129_ and also excludes the possibility that the activities observed in the DNA helicase assays are caused by putative contaminants in the protein preparations.

We conclude that PcrA^s^_M129_, PcrA^s^_FH_, PcrA*_Mpn_* and PcrA*_Mge_* each possess DNA helicase activity, and are capable of unwinding dsDNA substrates carrying either a 3′ or 5′ ss terminus. Thus, these proteins not only share sequence similarity with PcrA proteins from Gram-positive bacteria, but also in vitro DNA helicase activity.

### Reaction Requirements of the DNA Helicase Activities of the PcrA Proteins

As expected, the DNA helicase activity of the four PcrA proteins was found to be temperature-, Mg^2+^- and ATP-dependent (Fig. S2, and data not shown). Optimal activities of the proteins were observed at temperatures of 30–37°C, and at Mg^2+^ and ATP concentrations of 0.5–1 mM and 0.5–2.5 mM, respectively (Fig. S2).

**Figure 4:**
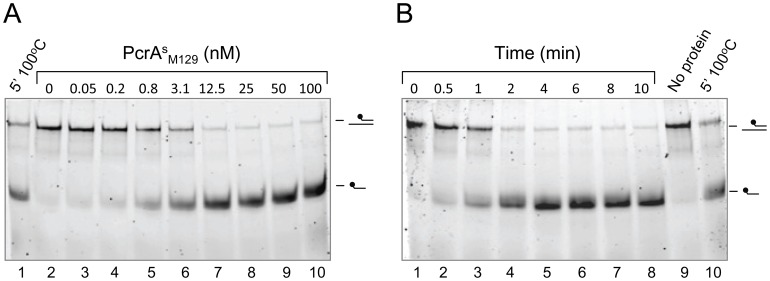
Protein concentration- and time-dependence of the DNA helicase activity of PcrA^s^_M129_. (A) Protein concentration-dependence of the DNA helicase activity. The DNA unwinding reactions were performed as described in Materials and Methods, using 4 nM of substrate ‘a’ and a range of PcrA^s^_M129_ concentrations (0–100 nM, as indicated above the lanes). Lane 1 shows a control reaction in which protein was omitted, and incubation was performed for 5 min at 100°C instead of at 37°C. (A) Time course of the DNA helicase activity of PcrA^s^_M129_ (at 0.1 µM) using substrate ‘a’ (at 4 nM). Reactions were performed at 37°C for the times indicated above the lanes. The control reactions shown in lanes 9 (‘No protein’) and 10 (‘5′ 100°C’) were incubated for 5 min at 37°C before deproteinization of the samples.

**Figure 5:**
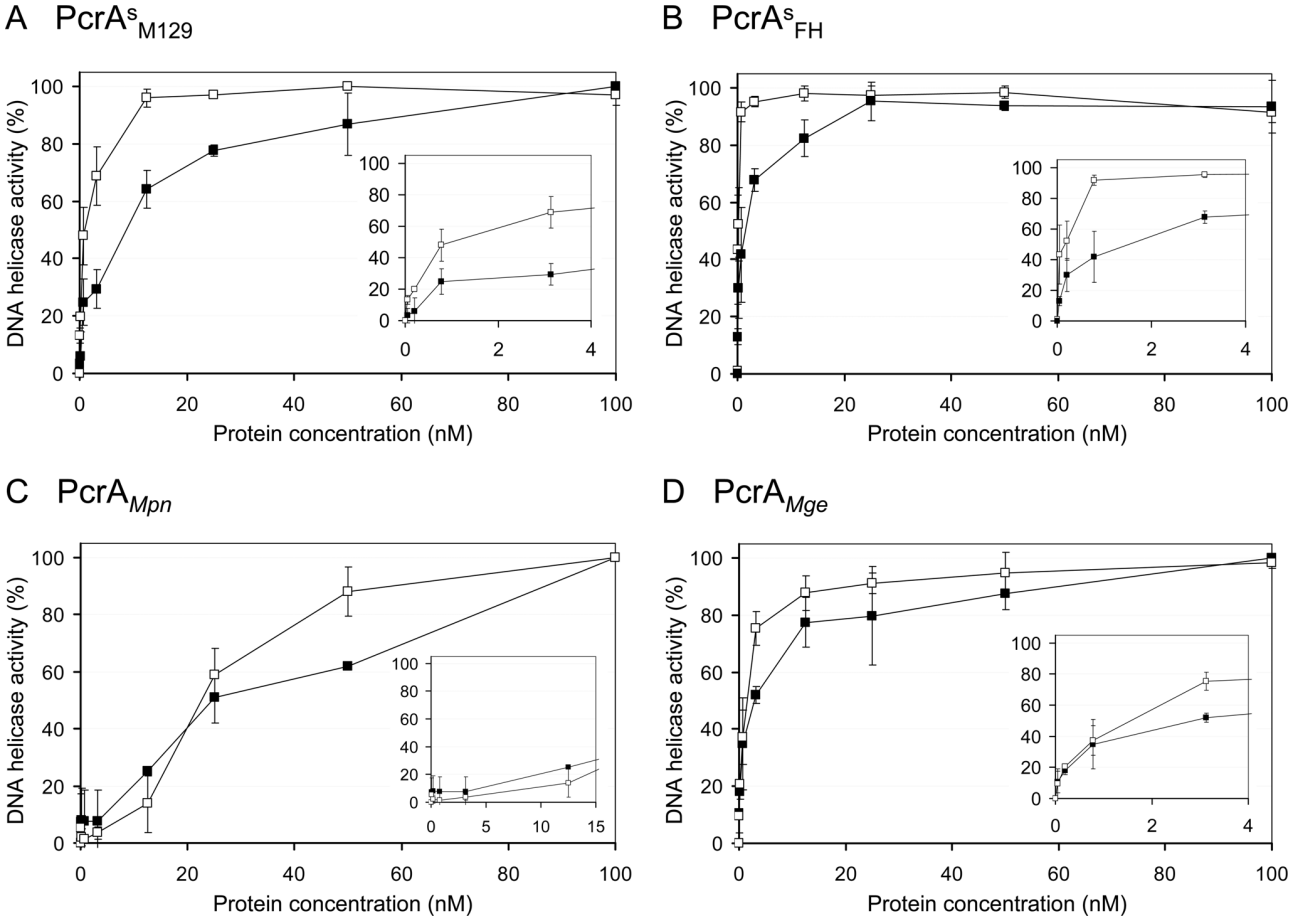
Protein concentration-dependence of the DNA helicase activities of the PcrA proteins. Reactions were carried out in a similar fashion as described in Fig. 4, and contained 4 nM of either substrate ‘a’ (□) or ‘b’ (▪) and a range of concentrations of either PcrA^s^_M129_ (A), PcrA^s^_FH_ (B), PcrA*_Mpn_* (C) or PcrA*_Mge_* (D). The protein concentrations tested were 0 pM, 50 pM, 0.2 nM, 0.8 nM, 3.2 nM, 12.5 nM, 25 nM, 50 nM and 100 nM. DNA unwinding was quantified by determination of the percentage of free (labeled) oligonucleotide that was displaced from the DNA substrates. Quantification was performed using Quantity One software (Bio-Rad), after separation of reaction products on polyacrylamide gels. Data shown are the average of two independent experiments. Error bars indicate the standard deviation of the mean. In the inset at the right-hand corner of each graph, a detail is shown of the DNA helicase activity (%) at the lowest protein concentrations tested.

**Figure 6:**
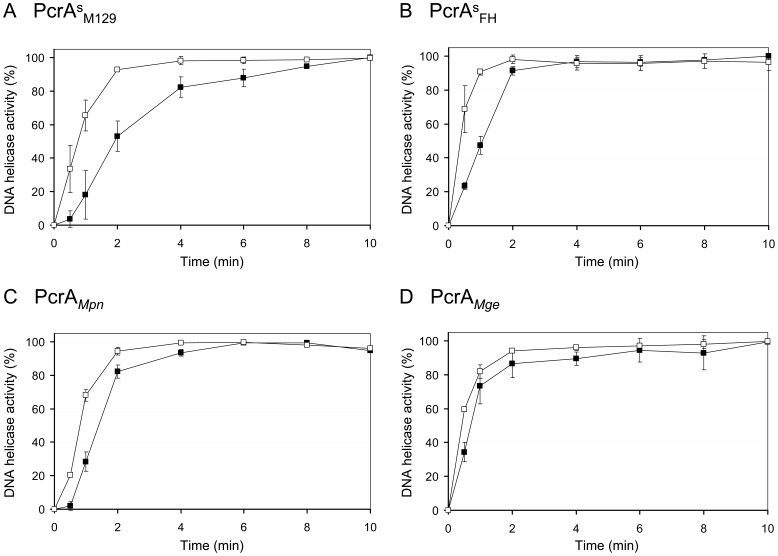
Time course of the DNA helicase activity of the PcrA proteins. DNA unwinding reactions were performed in a similar fashion as described in Fig. 4, and contained 4 nM of either substrate ‘a’ (□; see Fig. 1) or ‘b’ (▪) and 100 nM of either PcrA^s^_M129_ (A), PcrA^s^_FH_ (B), PcrA*_Mpn_* (C) or PcrA*_Mge_* (D). Reactions were terminated at either 0, 0.5, 1, 2, 4, 6, 8, or 10 min of incubation. Data were quantified and analyzed as described in the legend of Fig. 5.

The unwinding activity of PcrA^s^_M129_ on substrate ‘a’ (at 4 nM) could already be detected at a protein concentration of 0.8 nM, reaching optimal levels (>90% unwinding of the substrate) at concentrations ≥12 nM (Fig. 4A). Using 100 nM of PcrA^s^_M129_, optimal levels of unwinding of substrate ‘a’ (at 4 nM) were reached within 2 min of incubation (Fig. 4B and 5A). Similar characteristics were recorded for PcrA^s^_FH_ and PcrA*_Mge_* (Fig. 5 and Fig. 6). However, PcrA*_Mpn_* was required at a ∼4-fold higher concentration (∼50 nM) than the three other PcrAs in order to reach ∼90% unwinding of substrate ‘a’ within 5 min of incubation (Fig. 5C).

**Figure 7:**
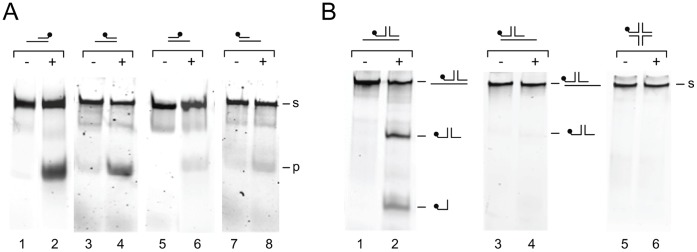
PcrA^s^_FH_ preferentially unwinds DNA substrates with a 3′ ss terminus. (A) DNA helicase activity is not significantly influenced by the position of the fluorescent label in the DNA substrate. DNA helicase assays were performed with PcrA^s^_FH_ and either substrate ‘a’ (6-FAM-labelled at either the 3′ end [lanes 1 and 2] or 5′ end [lanes 3 and 4] of strand 1 [Fig. 1]) or substrate ‘b’ (6-FAM-labelled at either the 3′ end [lanes 5 and 6] or 5′ end [lanes 7 and 8] of strand 1). The DNA helicase reactions were carried out in volumes of 10 µl and contained DNA substrate (at 8 nM) and either 0 nM (marked ‘−’, lanes 1, 3, 5 and 7) or 12 nM of protein (‘+’, lanes 2, 4, 6 and 8). After the reaction (10 min at 37°C), the samples were deproteinized, electrophoresed through a native 12% polyacrylamide gel, and analyzed by fluorometry. The positions of the substrates (s) and reaction products (p) are indicated at the right-hand side of the gels. (B) PcrA_s_^FH^ preferentially unwinds branched DNA substrates that carry a 3′ ss terminus. The DNA helicase activity of PcrA^s^_FH_ was tested on branched DNA substrates carrying either a 3′ ss terminus (substrate ‘d’, lanes 1 and 2), a 5′ ss terminus (substrate ‘e’, lanes 3 and 4) or blunt ends (substrate ‘f’, lanes 5 and 6). The sequences and structures of the different substrates are shown in Fig. 1. The reactions were performed in a similar fashion as described in (A).

From the time series experiment shown in Fig. 6, we estimated the time taken by the PcrA proteins to displace 50% of the DNA substrates. These data were subsequently converted to relative rates of DNA unwinding in a similar fashion as described by Soultanas and coworkers [48]. While PcrA^s^_FH_ was found to have the highest rate of DNA unwinding, considerably lower rates were observed for PcrA*_Mpn_* and PcrA^s^_M129_ (Table 2). It is also evident from Table 2 and Fig. 5 that each of the four proteins displayed a higher rate of unwinding of substrate ‘a’ than of substrate ‘b’. This indicated that substrates with a 3′ ss protruding end are more efficiently unwound by the PcrA proteins than substrates with a 5′ ss extension. This finding was not influenced by the position of the fluorescent label on the substrates, as substrates carrying a 3′ 6-FAM label where unwound with similar efficiencies as their 5′ 6-FAM-labeled counterparts (Fig. 7A).

**Table 2:** Relative rate of the DNA helicase activities of PcrA^s^_M129_, PcrA^s^_FH_, PcrA*_Mpn_* and PcrA*_Mge_*.

	Substrate ‘a’		Substrate ‘b’	
Protein	Time to unwind 50% of substrate (sec)	Relative rate (%)^a^	Time to unwind 50% of substrate (sec)	Relative rate (%)^a^
**PcrA^s^_M129_**	45.6	49	115.2	19
**PcrA^s^_FH_**	22.2	100	63.6	35
**PcrA** ***_Mpn_***	48.6	46	84.0	26
**PcrA** ***_Mge_***	25.2	88	42.6	52

a The data in Fig. 6 were fitted to estimate the time required to displace 50% of either substrate ‘a’ or substrate ‘b’. The results were then expressed as relative rates by comparison with the results obtained from the most efficient DNA helicase reaction, which included PcrA^s^_FH_ and substrate ‘a’. The procedure used to calculate the relative rates was described by Soultanas et al. [48].

The notion that DNA substrates with 3′ ss extensions are more efficiently unwound by the mycoplasmal PcrA proteins than substrates with either 5′ ss extensions or blunt ends, was corroborated by experiments in which additional oligonucleotide substrates were included. Specifically, a three-armed DNA substrate with a 3′ ss extension was unwound more efficiently than a similar substrate with a 5′ ss extension (Fig. 7B, compare lanes 2 and 4). Moreover, a branched DNA substrate carrying four ds ends was not detectably unwound by PcrA^s^_FH_ (Fig. 7B, lane 6), similar to what was reported above for the linear, blunt-ended substrate ‘c’ (Fig. 3G).

### The DNA Helicase Activity of the PcrA Proteins is Dependent on ATP Hydrolysis

As described above, the DNA unwinding activity of the four PcrA proteins was dependent on the presence of ATP in the reaction; in the absence of ATP, or in the presence of ATPγS, the proteins were inactive. Nevertheless, dATP could efficiently replace ATP as an essential nucleotide cofactor (Fig. S2). To investigate the ability of the PcrA proteins to consume ATP, an NADH-coupled ATPase assay was performed. As shown in Table 3, all four PcrA proteins were found to hydrolyze ATP. Interestingly, PcrA*_Mpn_* exhibited the highest ATPase rate of all four proteins (783.4±35.8 min^−1^ in the presence of ssDNA). Importantly, the ATPase rates were strongly induced by the presence of ssDNA in the reaction. While the basic ATPase rate of PcrA*_Mpn_* was stimulated ∼75-fold, the basic ATPase rates of the PcrA^s^ proteins were stimulated ∼30-fold by ssDNA. Only a ∼12-fold ssDNA-induced increase was observed in the ATPase rate of PcrA*_Mge_*. However, this protein displayed the highest basic rate of ATP hydrolysis (29.0±2.3 min^−1^) of all four proteins.

**Table 3:** ATPase rates of the PcrA helicases from *M. pneumoniae* and *M. genitalium*.

	ATPase rate^a±^SD (min^−1^) for:				
	MBP (negative control)	PcrA^s^_M129_	PcrA^s^_FH_	PcrA*_Mpn_*	PcrA*_Mge_*
**− ssDNA**	1.6±0.8	7.7±1.4	18.2±1.3	10.4±0.9	29.0±2.3
**+ ssDNA**	0.3±0.4	230.7±39.7	604.1±34.5	783.4±35.8	349.7±31.1
**Average stimulation** b	ND^c^	30.0×	33.2×	75.3×	12.1×

a The ATPase rates were determined either in the presence (+ ssDNA) or absence (- ssDNA) of φX174 virion DNA, as described in Materials and methods. The data represent averages (with standard deviation [SD]) from two independent measurements. Basal rates of ATP hydrolysis, which were determined in the absence of protein, were subtracted from the data.

b The average stimulation shows the average fold stimulation of the ATPase rate by ssDNA for each protein.

c ND, not determined.

## Discussion

The representatives of the *Mollicutes* class of bacteria have very compact genomes. Likewise, the set of genes encoding enzymes and proteins involved in the replication, recombination and repair of DNA is small in these organisms [4]–[6]. It was therefore surprising to find that the genome of *M. pneumoniae* harbors two consecutive genes that have the potential to encode PcrA-like DNA helicases, whereas *M. genitalium* only possesses one of such genes. In this study, we showed that both putative helicases of *M. pneumoniae*, i.e. PcrA^s^_M129_ (or PcrA^s^_FH_ in subtype 2 strains) and PcrA*_Mpn_*, possess Mg^2+^- and ATP-dependent DNA helicase activity. A similar activity could be attributed to the PcrA*_Mge_* protein from *M. genitalium*. Based on primary structure analysis as well as protein (sub)domain predictions, we proposed that PcrA*_Mge_* represents the ortholog of PcrA*_Mpn_*.

SF1 DNA helicases can be classified as SF1A or SF1B helicases [24], [34], [49]. Proteins belonging to the first group primarily have a 3′→5′ (or ‘type A’) polarity, whereas SF1B helicases have a 5′→3′ (‘type B’) polarity. A protein with a clear SF1A signature is the PcrA protein from *B. stearothermophilus* [50]. Other PcrA proteins, however, were shown to have a bipolar nature, by displaying similar helicase activities in the 3′→5′ and 5′→3′ directions. These proteins include PcrA*_Sau_* [45], PcrA*_Ban_* [43] and PcrA*_Spn_* [46]. The four PcrA proteins from *M. pneumoniae* and *M. genitalium* were also found to have bipolar activities, albeit that substrates with a 3′ ss extension were unwound somewhat more efficiently by these proteins than substrates with a 5′ ss extension.

Two of the mycoplasma PcrA proteins, i.e. PcrA*_Mpn_* and PcrA*_Mge_*, were found to resemble PcrA*_Bst_* also with respect to DNA binding characteristics; these proteins each prefer to bind to substrates containing ssDNA [50]. In contrast, PcrA*_Spn_*, PcrA*_Sau_* and PcrA*_Ban_* are unable to stably interact with ssDNA, and prefer to bind to substrates containing hairpins and/or partially ds regions [43], [45], [46], [50].

The most notable observations from this study, however, concern the characteristics of the PcrA^s^ proteins, which may be regarded as the first naturally occurring representatives of the SF1 family that lack an entire 2B subdomain. While the 2B subdomain sequences can vary significantly in size and sequence among SF1 helicases, the smallest 2B subdomains reported thus far are those of the HelD proteins from *E. coli* and *B. subtilis* (with lengths of 79 and 89 amino acids, respectively) [24], [34], [49], [51], [52]. Despite this apparent structural deficiency, both PcrA^s^ proteins were found to be highly active DNA helicases. However, this finding is not without precedent, because the dispensability of the 2B subdomain has previously been shown for another member of the SF1A protein family, Rep*_Eco_* [47]. In fact, a Rep*_Eco_* mutant deleted of the 2B region (RepΔ2B) displayed a faster rate of DNA unwinding than did the WT protein [47]. Based on this observation, it was suggested that the 2B subdomain might play a role in (i) (auto)regulation of the DNA helicase activity of Rep*_Eco_*, and (ii) the interaction with other, regulatory proteins [47], [53]. If this notion would also apply to the PcrA proteins from *M. pneumoniae*, this species would express one PcrA protein of which the activity can be regulated (PcrA*_Mpn_*), either intra- or intermolecularly, and a second PcrA protein that displays constitutive activity (PcrA^s^_M129_ or PcrA^s^_FH_).

Another unique property of the PcrA^s^ proteins as opposed to other PcrA proteins (including PcrA*_Mpn_* and PcrA*_Mge_*) was the inability to form stable protein-DNA complexes in EMSA. It is possible that this deficiency is the consequence of the lack of a 2B subdomain, and that this subdomain plays a role in stable DNA binding. In agreement with this notion, the 2B subdomain from PcrA*_Bst_* was found to interact with the ds part of a small DNA substrate with a 3′ ss tail in PcrA*_Bst_*-DNA crystal structures [36]. The introduction of specific point mutations in the 2B region of PcrA*_Bst_* resulted in proteins (K419A, T426A and K456A) that were defective in dsDNA binding and helicase activity [48].

A crucial question that remains to be addressed is the in vivo role of the mycoplasmal PcrA DNA helicases. As mentioned above, the PcrA proteins from *B. subtilis* and *S. aureus* are essential for cell growth and viability [25]–[27]. Moreover, PcrA*_Bsu_* was shown to restore UV resistance in a *uvrD* mutant of *E. coli*, and to play a role in the resolution of stalled replication forks [25], [26]. Interestingly, the lethality of a *pcrA* null mutation in *B. subtilis* could be suppressed by additional mutations in genes *recF*, *recL*, *recO* and *recR*, which belong to the same complementation group [26]. While the function of RecL is unknown, the RecF, RecO and RecR (RecFOR) proteins assist the major recombinase RecA in binding to ssDNA, and thereby initiate and catalyze homologous DNA recombination. Thus, the lethality of the *pcrA* null mutant is an indirect phenomenon, which can be overcome by inactivation of the RecFOR-dependent recombination pathway. It was suggested that RecFOR induces an unusually high (and thereby toxic) level of recombination when PcrA is absent. This notion was supported by the observation that *B. subtilis* strains become hyper-recombinogenic (∼15 times higher than the WT strain) when the amount of PcrA is reduced by a factor of 10 [26]. This high level of recombination was dependent on both RecA and the RecFOR pathway [26]. Thus, PcrA*_Bsu_* appears to have an anti-recombinogenic effect.

In contrast to the situation in Gram-positive bacteria, the PcrA proteins are not essential in *M. pneumoniae* and *M. genitalium*. By using a global transposon mutagenesis protocol, several *pcrA* transposon insertion mutants of *M. genitalium* were obtained [54]. In addition, we recently identified *M. pneumoniae* M129 mutant strains carrying transposon insertions in either ORF MPN340 or MPN341 (E. Spuesens, C. Vink, J. Stülke, unpublished data). The non-essential nature of these genes in *M. pneumoniae* and *M. genitalium* is not surprising, however, because (i) the lethality of the *pcrA* genes in Gram-positive bacteria is dependent on a functional RecFOR pathway, and (ii) the RecFOR pathway is absent in *M. pneumoniae* and *M. genitalium* [6], [17]. This raises the question why PcrA function is maintained in these mycoplasmas during evolution, whereas RecFOR has been lost.

Another crucial question is whether the PcrA proteins from *M. pneumoniae* and *M. genitalium* have a similar anti-recombinogenic function as do their gram-positive counterparts, despite the absence of a RecFOR pathway in the mycoplasmas. In this regard, it is tempting to speculate on a putative role of the PcrA proteins in homologous recombination between repetitive DNA elements in *M. pneumoniae* and *M. genitalium.* These recombination processes were found to induce antigenic variation of major bacterial surface proteins (for a review, see [4]). It was shown for *M. genitalium* that these events depend upon the function of the RecA protein [55]. Interestingly, the frequency of recombination between repetitive DNA elements is higher in *M. genitalium* than in *M. pneumoniae*. This difference was previously hypothesized to be caused by differences in the specific activities of the RuvA, RuvB and RecU proteins from these species [4], [14], [16], [17]. However, it is also possible that the *M. pneumoniae* PcrA^s^ proteins, which do not have an ortholog in *M. genitalium*, play an inhibitory role in the recombination between repetitive DNA elements. This notion is currently being tested by monitoring (the changes in) the sequences of the repetitive elements during propagation of the *M. pneumoniae* MPN340 null mutant in culture.

The involvement of an SF1 family member in DNA recombination-induced antigenic variation is not unprecedented. In Gram-negative bacterium *Neisseria gonorrhoeae*, a system of antigenic variation is operational that is similar to that in *M. genitalium* and *M. pneumoniae*. This system, which is termed pilin antigenic variation, depends on the function of a set of proteins that not only includes RecA, RuvA, RuvB, and RuvC, but also Rep [4], [56]. However, in contrast to the suggested negative effect of PcrA^s^ on homologous DNA recombination in *M. pneumoniae*, the Rep protein of *N. gonorrhoeae* was found to have a positive influence on the overall efficiency of pilin antigenic variation [56].

Finally, it is important to consider that this study was performed exclusively with MBP-fused proteins. We were constrained to use these fusion proteins because the native versions of the PcrA proteins were either expressed at very low levels, or could not be purified in their native state due to solubility problems. While the MBP tag may theoretically influence the activity of the attached protein, numerous studies are available showing that this tag is functionally inert, in particular concerning the function of DNA-interacting fusion partners, such as the integrase proteins from HIV-1, HIV-2 and feline immunodeficiency virus [28], [57], the *M. genitalium* HJ resolvase RecU*_Mge_* [14], [15], the *E. coli* HJ resolvase RuvC [58] and the RuvB helicases from *M. pneumoniae* and *M. genitalium* [16]. Moreover, several other SF1 proteins have previously been shown to be fully active as variants containing an N-terminal polyhistidine-tag. These proteins include PcrA*_Ban_* [43], [44], PcrA*_Bce_* [44], PcrA*_Sau_* [20], [45], [59] and PcrA*_Spn_* [46]. Also, it was shown that the activities of a Rep*_Eco_* mutant, RepΔ2B, differed only marginally (in efficiency) from those of an N-terminally tagged variant of this protein (+HRepΔ2B) [47]. Nonetheless, attempts to obtain non-tagged variants of the mycoplasma PcrA proteins are ongoing in our laboratory.

In conclusion, we have determined the in vitro activities of the SF1 proteins encoded by *M. pneumoniae* and *M. genitalium*, and found each of these proteins to act as DNA helicases in vitro. The main challenge of future studies will be to determine the in vivo roles of these proteins, in particular in light of the lack of a RecFOR pathway in both *M. pneumoniae* and *M. genitalium*. We will also aim to address the question if, and how, the two different PcrA proteins from *M. pneumoniae* interact, either physically or functionally. Clearly, the answers to these questions will shed further light on the functionalities of the DNA repair and recombination pathways in bacteria with a strongly reduced, or ‘minimal’ [54], genome.

## Supporting Information

Figure S1 **Multiple alignment of the amino acid sequences of the PcrA(-like) proteins from** ***M. pneumoniae*** **and** ***M. genitalium*** **.** (A) An alignment was generated with amino acid sequences predicted to be encoded by the following ORFs (with UniProtKB numbers in parentheses): MPN340 from *M. pneumoniae* strain M129 (P75438; PcrA^s^_M129_); MPN_0394 from *M. pneumoniae* strain FH (E1QC92; PcrA^s^_FH_); MPN341 from *M. pneumoniae* strain M129 (P75437; PcrA*_Mpn_*); MG244 from *M. genitalium* strain G37 (P47486; PcrA*_Mge_*); *pcrA* from *Lactobacillus salivarius* strain UCC118 (Q1WSH5; PcrA*_Lsa_*); *pcrA* from *Staphylococcus aureus subsp. aureus* strain MSHR1132 (G7ZPU1; PcrA*_Sau_*). Predicted domains and motifs of the PcrA(-like) proteins are indicated above and below the alignment and are predominantly based on the crystal structure of the PcrA protein from *Bacillus stearothermophilus* [35]. The multiple alignment was performed using Clustal W (http://www.ebi.ac.uk/Tools/msa/clustalw2/). The program BOXSHADE 3.21 (http://www.ch.embnet.org/software/BOX_form.html) was used to produce white letters on black boxes (for amino acid residues that are identical in at least three out of six sequences) and white letters on grey boxes (for similar residues). The three residues that differ between PcrA^s^_M129_ and PcrA^s^_FH_ are indicated by red dots above the sequences.
(TIF)

Figure S2 **Reaction requirements of the DNA helicase activity of PcrA** ***_Mge_*** **.** (A) Temperature-dependence of the DNA helicase activity of PcrA*_Mge_*. Reactions were carried out for 5 min at either 0°C (lane 1), 10°C (lane 2), 20°C (lane 3), 30°C (lane 4), 37°C (lane 5) or 45°C (lane 6) in the presence of substrate ‘a’ (8 nM) and 80 nM of PcrA*_Mge_*. Lane 7 shows a control reaction that was incubated at 37°C in the absence of protein. The reaction shown in lane 8 was performed in the absence of protein for 5 min at 100°C (instead of at 37°C). (B) Mg^2+^-dependence of the DNA helicase activity of PcrA*_Mge_*. Reactions were performed at various concentrations of Mg^2+^ (as indicated above the lanes), in the presence of substrate ‘a’ (8 nM) and 80 nM of PcrA*_Mge_*. (C) ATP-dependence of the DNA helicase activity of PcrA*_Mge_*. Reactions were performed at various concentrations of ATP (as indicated above the lanes) in the presence of substrate ‘a’ (8 nM) and 80 nM of PcrA*_Mge_*. (D) Nucleotide cofactor-dependence of the DNA helicase activity of PcrA*_Mge_*. Reactions contained substrate ‘a’ (8 nM), 1 mM MgCl_2_ and 80 nM PcrA*_Mge_*, and were performed in the absence (lane 1; ‘No ATP’) or presence of 1 mM of either ATP (lane 2), ATPγS (lane 3), dATP (lane 4), dCTP (lane 5), dGTP (lane 6) or dTTP (lane 7).
(TIF)

Table S1 Oligonucleotide primers used for the cloning of ORFs encoding the PcrA-like helicases from *M. pneumoniae* and *M. genitalium.* ^a^The different ORFs are from *M. pneumoniae* strains M129 (MPN340 and MPN341) and FH (MPNE_0394), and from *M. genitalium* strain G37 (MG244). ^b^Restriction endonuclease recognition sites that were incorporated in the primer sequences for cloning purposes are indicated in italics. The TGG codons (or the complementary sequences CCA) that were incorporated in the oligonucleotides in order to modify the TGA codons within the native ORFs, are underlined.
(DOC)
