# Supplementary figures and images for: Functional Analysis of the Superfamily 1 DNA Helicases Encoded by Mycoplasma pneumoniae and Mycoplasma genitalium
Source: PLoS One. 2013 Jul 23;8(7):e70870. doi: 10.1371/journal.pone.0070870 (PMC3720892; doi:10.1371/journal.pone.0070870)

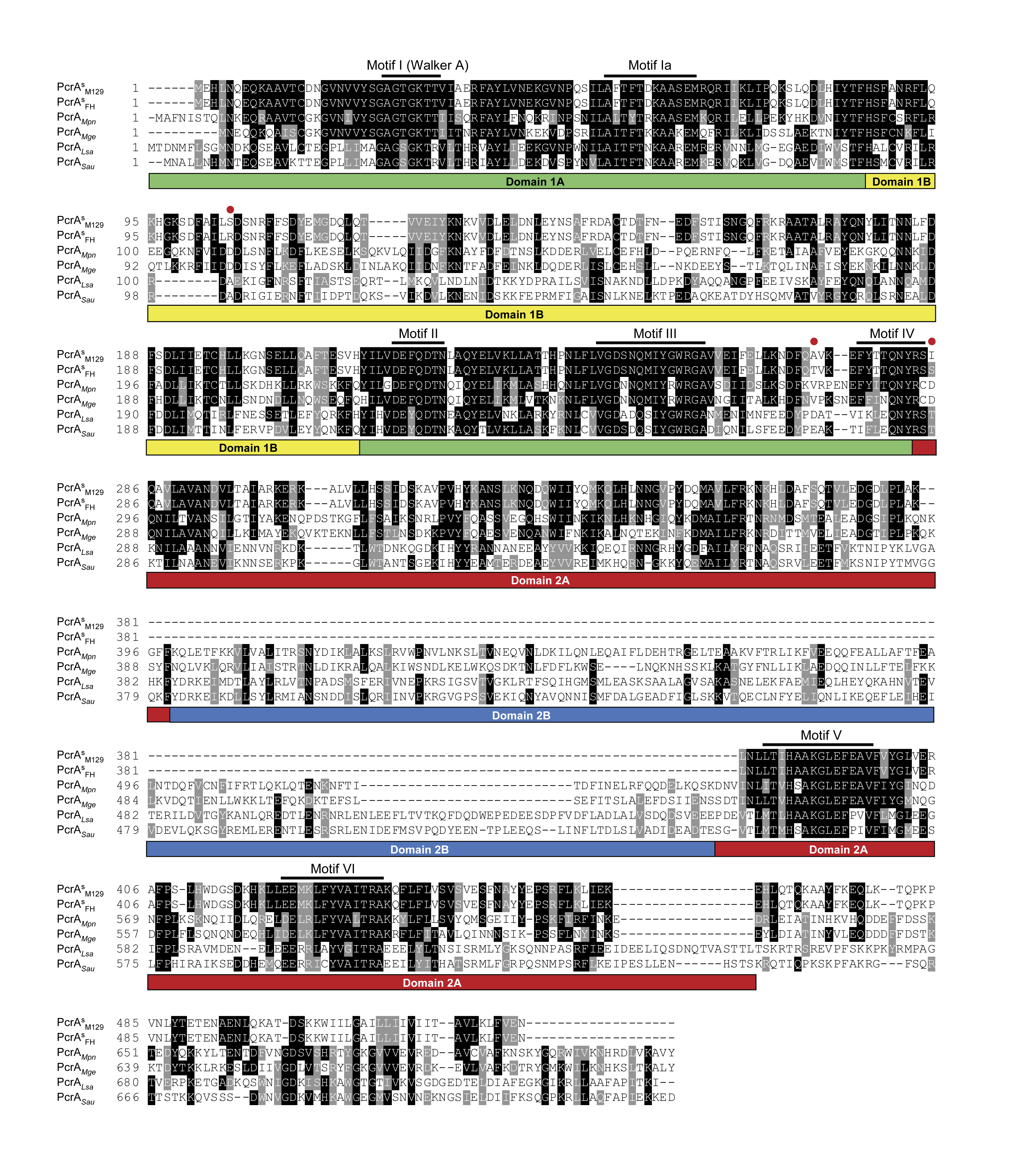

Supplement: Figure S1 — Multiple alignment of the amino acid sequences of the PcrA(-like) proteins from M. pneumoniae and M. genitalium . (A) An alignment was generated with amino acid sequences predicted to be encoded by the following ORFs (with UniProtKB numbers in parentheses): MPN340 from M. pneumoniae strain M129 (P75438; PcrAs M129); MPN_0394 from M. pneumoniae strain FH (E1QC92; PcrAs FH); MPN341 from M. pneumoniae strain M129 (P75437; PcrAMpn); MG244 from M. genitalium strain G37 (P47486; PcrAMge); pcrA from Lactobacillus salivarius strain UCC118 (Q1WSH5; PcrALsa); pcrA from Staphylococcus aureus subsp. aureus strain MSHR1132 (G7ZPU1; PcrASau). Predicted domains and motifs of the PcrA(-like) proteins are indicated above and below the alignment and are predominantly based on the crystal structure of the PcrA protein from Bacillus stearothermophilus [35]. The multiple alignment was performed using Clustal W (http://www.ebi.ac.uk/Tools/msa/clustalw2/). The program BOXSHADE 3.21 (http://www.ch.embnet.org/software/BOX_form.html) was used to produce white letters on black boxes (for amino acid residues that are identical in at least three out of six sequences) and white letters on grey boxes (for similar residues). The three residues that differ between PcrAs M129 and PcrAs FH are indicated by red dots above the sequences. (TIF) [file pone.0070870.s001.tif]

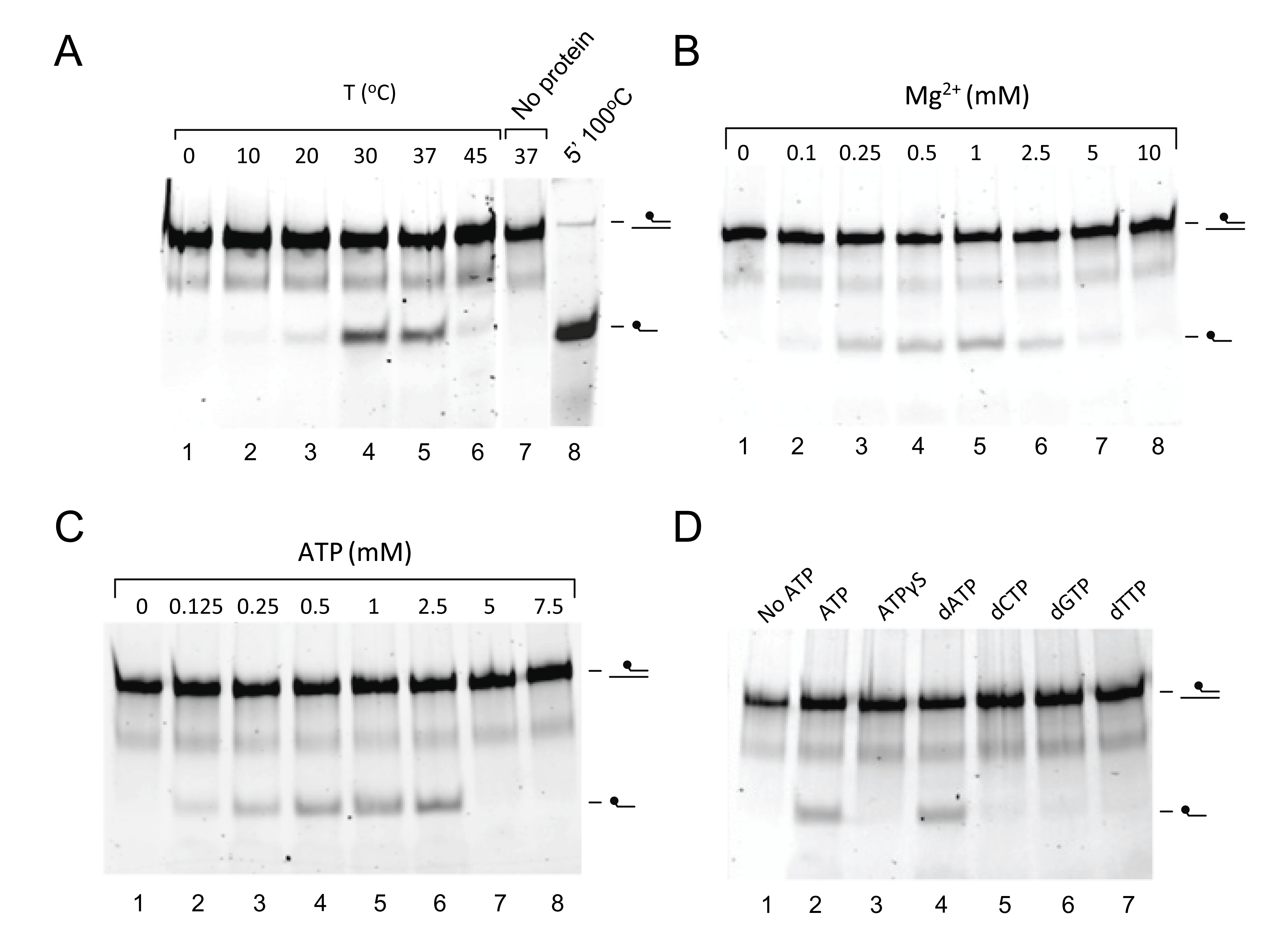

Supplement: Figure S2 — Reaction requirements of the DNA helicase activity of PcrA Mge . (A) Temperature-dependence of the DNA helicase activity of PcrAMge. Reactions were carried out for 5 min at either 0°C (lane 1), 10°C (lane 2), 20°C (lane 3), 30°C (lane 4), 37°C (lane 5) or 45°C (lane 6) in the presence of substrate ‘a’ (8 nM) and 80 nM of PcrAMge. Lane 7 shows a control reaction that was incubated at 37°C in the absence of protein. The reaction shown in lane 8 was performed in the absence of protein for 5 min at 100°C (instead of at 37°C). (B) Mg2+-dependence of the DNA helicase activity of PcrAMge. Reactions were performed at various concentrations of Mg2+ (as indicated above the lanes), in the presence of substrate ‘a’ (8 nM) and 80 nM of PcrAMge. (C) ATP-dependence of the DNA helicase activity of PcrAMge. Reactions were performed at various concentrations of ATP (as indicated above the lanes) in the presence of substrate ‘a’ (8 nM) and 80 nM of PcrAMge. (D) Nucleotide cofactor-dependence of the DNA helicase activity of PcrAMge. Reactions contained substrate ‘a’ (8 nM), 1 mM MgCl2 and 80 nM PcrAMge, and were performed in the absence (lane 1; ‘No ATP’) or presence of 1 mM of either ATP (lane 2), ATPγS (lane 3), dATP (lane 4), dCTP (lane 5), dGTP (lane 6) or dTTP (lane 7). (TIF) [file pone.0070870.s002.tif]
