# Supplementary material for: Functional Analysis of the Superfamily 1 DNA Helicases Encoded by Mycoplasma pneumoniae and Mycoplasma genitalium
Source: PLoS One. 2013 Jul 23;8(7):e70870. doi: 10.1371/journal.pone.0070870 (PMC3720892; doi:10.1371/journal.pone.0070870)
Supplement: Table S1 — Oligonucleotide primers used for the cloning of ORFs encoding the PcrA-like helicases from M. pneumoniae and M. genitalium. aThe different ORFs are from M. pneumoniae strains M129 (MPN340 and MPN341) and FH (MPNE_0394), and from M. genitalium strain G37 (MG244). bRestriction endonuclease recognition sites that were incorporated in the primer sequences for cloning purposes are indicated in italics. The TGG codons (or the complementary sequences CCA) that were incorporated in the oligonucleotides in order to modify the TGA codons within the native ORFs, are underlined. (DOC) [file pone.0070870.s003.doc]

**Table S1.** Oligonucleotide primers used for the cloning of ORFs encoding the PcrA-like helicases from *M. pneumoniae* and *M. genitalium.*

| **ORF^a^** | **Primer name** | **Sequence (5’>3’)^b^** |
| --- | --- | --- |
| **MPN340 and MPNE_0394** |  |  |
|  | pET_Fw | GATCG*CATATG*GAACATTTAAACAGGA |
|  | pET_Rv | CAGTT*GGATCC*TAAATGCCATAGTACTAGGT |
|  | pMAL-c_Fw | GATCG*GAATTC*ATGGAACATTTAAATCAGGA |
|  | pMAL-c_Rv | CAGTT*CTGCAG*TAAATGCCATAGTACTAGGT |
|  | Mutation_Fw | TGACTCTAAAAAGTGGATTATTTTAGGGGCG |
|  | Mutation_Rv | CGCCCCTAAAATAATCCACTTTTTAGAGTCA |
|  | 340FW_K>R | GTGCTGGCACGGGTAGAACAACCGTTATTGC |
|  | 340RV_K>R | GCAATAACGGTTGTTCTACCCGTGCCAGCAC |
|  | 340FW_K>A | GTGCTGGCACGGGTGCAACAACCGTTATTGC |
|  | 340RV_K>A | GCAATAACGGTTGTTGCACCCGTGCCAGCAC |
| **MPN341** |  |  |
|  | 341pETfw | GCATT*CATATG*GCATTTAACATTAGCAC |
|  | 341pETrv | GATAC*GGATCC*CTTTTCCATGATTATAGTGA |
|  | 341pMALcfw | GCATT*TCTAGA*ATGGCATTTAACATTAGCAC |
|  | 341pMALcrv | GATAC*CTGCAG*CTTTTCCATGATTATAGTGA |
|  | 341Mut1 | CAAATTATTGCGCAAGTGGTCGAAAAAATT |
|  | 341Mut2 | GATGATTTACCGCTGGAGGGGCGCG |
|  | 341Mut3 | GCCAACATTCTTGGATTATCAATAAGATTA |
|  | 341Mut4 | GTTTGCGGGTTTGGCCTAATGTTTTAAA |
|  | 341Mut5fw | GTACGGACAGAGGTGGATTGTTAAAAATCAC |
|  | 341Mut1rv | AATTTTTTCGACCACTTGCGCAATAATTTG |
|  | 341Mut2rv | CGCGCCCCTCCAGCGGTAAATCATC |
|  | 341Mut3rv | TAATCTTATTGATAATCCAAGAATGTTGGC |
|  | 341Mut4rv | TTTAAAACATTAGGCCAAACCCGCAAAC |
|  | 341Mut5rv | GTGATTTTTAACAATCCACCTCTGTCCGTAC |
| **MG244** |  |  |
|  | 244pETfw | GAATC*CATATG*AATGAACAACAAAAACA |
|  | 244pETrv | GTCAT*GGATCC*AAGATCATTTAGTTCTGCTC |
|  | 244pMALcfw | GAATC*GGATCC*ATGAATGAACAACAAAAACA |
|  | 244pMALcrv | GTCAT*CTGCAG*AAGATCATTTAGTTCTGCTC |
|  | 244Mut1 | GATTTACCGCTGGAGAGGGGCGGT |
|  | 244Mut2-3 | CCCTAAAAATTTGGTCAAATGATTTAAAGGAATTGTGGAAACAGAG |
|  | 244Mut4 | GATTTTCTTAAATGGTCAGAATTAAATC |
|  | 244Mut5 | GAAAATCTGCTTTGGAAAAAACTAACTG |
|  | 244Mut1rv | ACCGCCCCTCTCCAGCGGTAAATC |
|  | 244Mut2-3rv | CTCTGTTTCCACAATTCCTTTAAATCATTTGACCAAATTTTTAGGG |
|  | 244Mut4rv | GATTTAATTCTGACCATTTAAGAAAATC |
|  | 244Mut5rv | CAGTTAGTTTTTTCCAAAGCAGATTTTC |

^a^The different ORFs are from *M. pneumoniae* strains M129 (MPN340 and MPN341) and FH (MPNE_0394), and from *M. genitalium* strain G37 (MG244).

^b^Restriction endonuclease recognition sites that were incorporated in the primer sequences for cloning purposes are indicated in italics. The TGG codons (or the complementary sequences CCA) that were incorporated in the oligonucleotides in order to modify the TGA codons within the native ORFs, are underlined.
